# Structural investigation of Ayurveda Lauha (Iron) Bhasma

**DOI:** 10.1016/j.jaim.2023.100690

**Published:** 2023-02-21

**Authors:** M.K. Tiwari, Arjun Singh, Ajay Khooha, U.K. Goutam

**Affiliations:** a Accelerator Physics and Synchrotrons Utilization Division, Raja Ramanna Centre for Advanced Technology, Indore, 452013, India; b Homi Bhabha National Institute, Anushaktinagar, Mumbai, 400094, India; c Central Council for Research in Ayurvedic Sciences, Ministry of AYUSH, New Delhi, 110058, India; d Technical Physics Division, Bhabha Atomic Research Centre, Mumbai, 400085, India

**Keywords:** Ayurvedic Bhasma, Nanomaterials, X-ray fluorescence, Trace element analysis, Synchrotron radiation

## Abstract

In Ayurveda, ‘Lauha’ (Iron) Bhasma is primarily used to cure diseases related to iron deficiency in humans. It is produced from purified raw metallic iron using a combination of multi-step traditional preparation processes described in the Ayurveda literature. Here, we present the results of structural investigation performed on the medicinal grade ‘Lauha’ Bhasma using various X-ray based techniques. Our results indicate that after several rounds of heating and cooling in specific conditions following the Ayurvedic preparation procedure, metallic iron eventually converts to a natural iron-oxide mineral belonging to the magnetite group. Scanning electron microscopy (SEM) and X-ray standing wave assisted fluorescence measurements carried out on powdered Bhasma specimen reveal that the magnetite micro-particles in the Bhasma specimen are usually present in the form of agglomerates of nano-particles. We anticipate that the Ayurvedic Lauha Bhasma has great potential for noninvasive localized target killing of cancer cells, particularly in sensitive parts of the human body such as the brain, spinal cord, and lungs, via necrosis by application of an alternating external magnetic field or photo electron generation through X-rays.

## 1 Introduction

Ayurveda is a traditional medicinal system of India that was evolved after generating comprehensive understanding about the anatomy of a human body over centuries of experience in the ancient past. This system was quite popular and was practiced routinely in India by certified Ayurveda practitioners for a long period of time until the nineteenth century, whilst modern Allopathic system medicine had not fully evolved [1,2]. Ayurvedic medicines are commonly thought to be those that are derived or synthesised from specific plants that have medicinal properties, such as turmeric (*Curcuma longa*), Brahmi (*Bacopa monnieri*), ghrtakumaaree (*Aloe barbadensis miller*), giloy (*Tinospora cordifolia*), Ashwagandha (*Withania somnifera*), Sunthi (*Zingiber officinale*), etc. There are hundreds of such plants that are especially utilised to extract compounds from their different parts (e.g., root, stalk, leaf, flower, and nut), which offer various medicinal properties.

In another branch of Ayurveda medicinal system which is usually referred to ‘*Rasa Shastra*’, certain minerals and metallic based compounds (*Bhasma*) [3] are commonly utilised to treat or cure various diseases of the human body. Ayurvedic Bhasma are herbo-metallic ashes which are prepared through calcination process by mixing particular combination of metal complexes along with various plants and biogenic-based ingredients. It is anticipated that these metal complexes [4] should not contain any free components of the original base metal or organic constituents employed during the various processes of their synthesis. The presence of any primary contents in their raw form in a Bhasma specimen is considered as improper calcination process in Ayurveda and is subjected for rejection. A medicinal-grade Ayurveda Bhasma must qualify certain specific criteria, which are detailed elsewhere [5]. Various herbo-minerals used in Ayurveda medicines offer them unique therapeutic properties such as low dose needed for treatment (*Alpamatropayogitvat*), good palatability (*Arucher-aprasangata*) and rapid response (*kshipramar-ogayadayitvat*) to a disease. It is expected that ∼35–40% of Ayurvedic medicines listed in the Ayurveda formulary contains at least one metal complex [[6], [7], [8], [9]].

Iron is an essential element for almost all living species on our planet, from bacteria to mammals. It effectively controls a wide range of metabolic functions in our body, including oxygen transport and the synthesis of deoxyribonucleic acid [10,11]. The importance of the Fe element resides in its ability to control electron transport phenomena. In the ferrous state (Fe^2+^), it behaves as an electron donor, whereas in the ferric state (Fe^3+^) it acts as an electron acceptor. As a result, it plays a vital role as a catalyst in a variety of enzymatic reactions involving electron transport phenomena. It is known that our bodies regularly lose iron in small amounts through urination, defecation, sweating, sloughing-off skin cells, and particularly menstrual bleeding in women. Hence, to maintain proper biological functions, it is important to maintain an adequate amount of iron intake through food that can reproduce hemoglobin as well as other important bio-molecules required in our bodies.

In the ancient past, in the Ayurvedic medicinal system, acute iron deficiency (anemia), liver enlargement, and jaundice in humans were primarily treated through digestion of ‘Lauha Bhasma’ produced from wrought iron as well as naturally occurring iron oxide minerals. Although at present many iron-based compounds are frequently used in the modern allopathic medicinal system to treat anemia in humans [12], however the use of ‘Lauha Bhasma’ is still popular in the Indian subcontinent as a therapeutic agent for the aforementioned diseases [13]. It is imperative to understand how Bhasma materials interact with human bodies and treat severe diseases without causing adverse effects. Such unique features of Ayurvedic medicines are deeply correlated with their structural, chemical, and physical properties, which have not been thoroughly recognized so far in the context of modern perspectives. X-ray investigations of these materials make it possible to investigate potential correlations, if any exist, between their physico-microstructural and therapeutic properties.

Here, we present structural and chemical investigations of ‘Lauha Bhasma’ using different X-ray techniques such as X-ray diffraction (XRD), X-ray fluorescence (XRF), and X-ray photoelectron spectroscopy (XPES) using intense X-rays produced from a synchrotron source. Our results demonstrate that iron is essentially present in the magnetite (Fe_3_O_4_) phase in the medicinal grade ‘Lauha Bhasma’. The XRF and XPES investigations further indicate that Fe occurs in the Lauha Bhasma mostly in the pure oxide phase along with trace amounts of C, Na, and Zn elements present as contaminants. Scanning electron microscopy (SEM), optical, and X-ray standing wave-assisted measurements have also revealed that Fe_3_O_4_ exists in the form of fine granular particles composed of agglomerates of nanoparticles with sizes 100 nm and larger. Our results support some of the previous studies done on the ‘Lauha Bhasma’, which was prepared using different modern and traditional methods reported in the Ayurveda literature [[14], [15], [16], [17]].

## 2 Material and methods

In early days, according to *Rasa Ratna Samuchchhaya*, a naturally occurring magnetite iron ore (Kanta Lauha) was considered the material of choice for the preparation of Lauha Bhasma [14]. Now a days, turnings of wrought iron (Teekshna Lauha) are extensively used for the preparation of Lauha Bhasma in the Ayurveda pharmaceutical industry. An elaborated process has already been reported in the literature [15] for the preparation of Lauha Bhasma, considering clax of Fe turnings, as a starting material. It may be important to mention that the careful preparation procedure plays an important role in deciding the therapeutic efficacy of the Lauha Bhasma.

In the present study, medicinal grade Lauha Bhasma was synthesized from Teekshna Lauha (clax of Fe turnings) after employing various preparation steps as described in the Ayurveda formulary [16] such as *samanya shodhan* (normal purification), *vishesha sodhan* (special purification), Bhanupaka (exposure to sun light) and putapaka (calumniation). At the end of Bhasma preparation procedure, various physico-chemical analysises such as color, texture, and floating test on the water surface was carried out to evaluate its properties according to the Ayurveda formulary before animal trials [18].

## 3 Results

### 3.1 Structural analysis

**Fig. 1:**
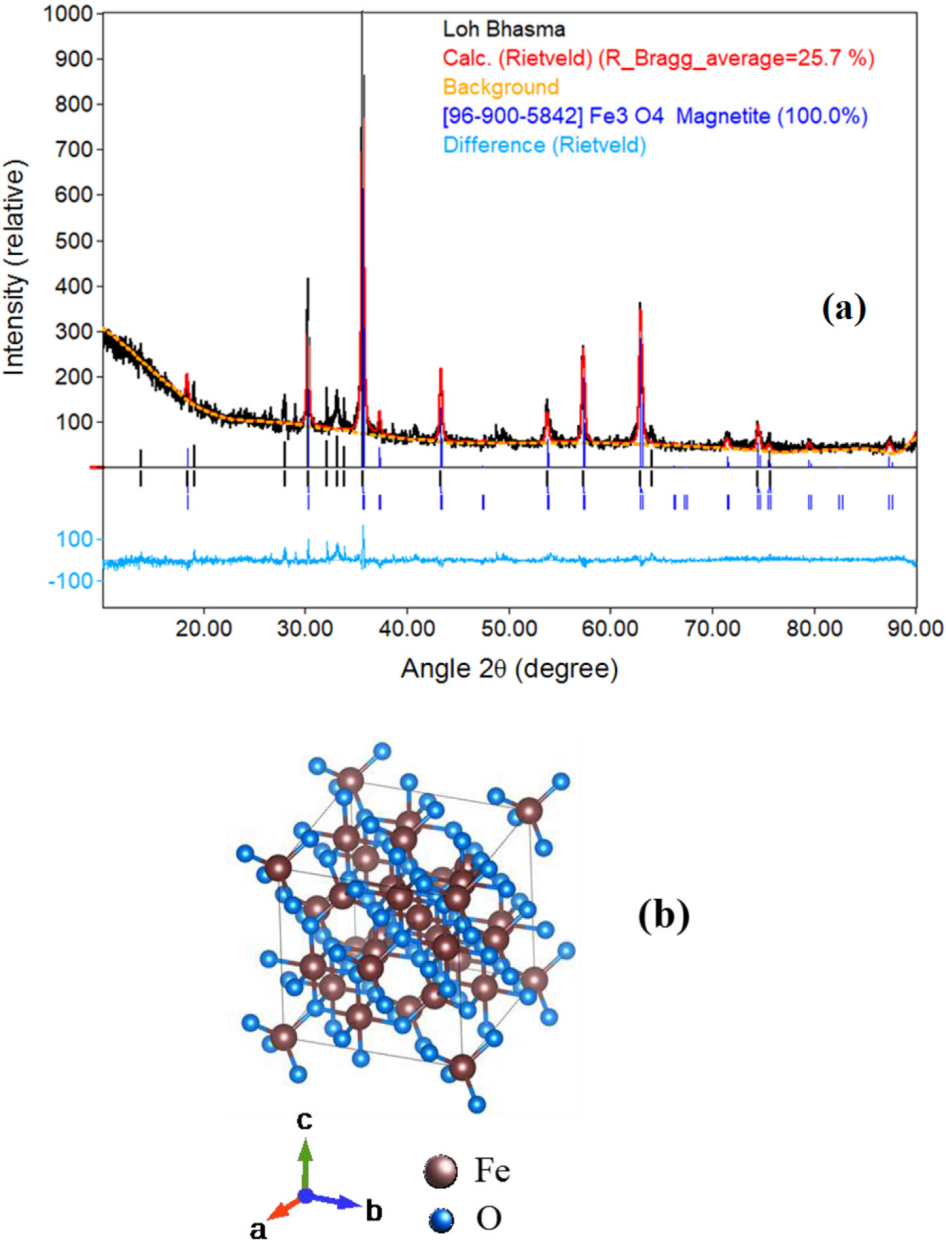
(a) Measured XRD pattern of the medicinal grade Lauha Bhasma sample along with fitted spectrum. (b) atomic arrangement in a magnetite structure calculated using Rietveld analysis.

X-ray diffraction (XRD, D8 Advance, BRUKER) measurements were carried out on the medicinal grade Lauha Bhasma to obtain its detailed structural properties and mineralogical information. The XRD pattern was recorded using a laboratory-based instrument at Cu-Kα X-ray energy (λ = 1.54 Å). The XRD pattern of Lauha Bhasma powder was recorded by varying the diffraction angle (2θ) in the range of 10°–90°. During the measurement, the sample was continuously rotated in the horizontal plane to collect information about all possible crystalline planes that exist in the powdered Bhasma specimen. Fig. 1a depicts the measured XRD spectrum of the Fe Bhasma sample. The initial fitting of the XRD data was carried out using the FullProf software [19]. Standard JCPDS data of different iron oxide structures were considered during the Rietveld analysis.

The detailed Rietveld analysis of the measured XRD spectrum indicates that the pharmaceutical grade Fe Bhasma is primarily composed of the Fe_3_O_4_ magnetite phase. It was also noticed that some of the XRD peaks especially in the angular range of 32°–38°, do not perfectly match with the fitted Rietveld pattern. This suggests that in addition to the Fe_3_O_4_ compound, the Fe Bhasma may also contain some other mixed compound phases in trace quantities, which are expected to form due to contamination with low atomic number impurity elements or various bio-herbal ingredients (carbon-based compounds), used during the Bhasma preparation procedure. Fig. 1b shows the crystal structure of magnetite Fe_3_O_4_ compound derived from the Rietveld analysis. It was found to closely agree with the published literature [[20], [21], [22]]. The average crystallite size in the Lauha Bhasma was also determined using Williamson-Hall (W–H) analysis based on the observed broadening of different XRD peaks. It was estimated to be ∼12.2 Å.

**Fig. 2:**
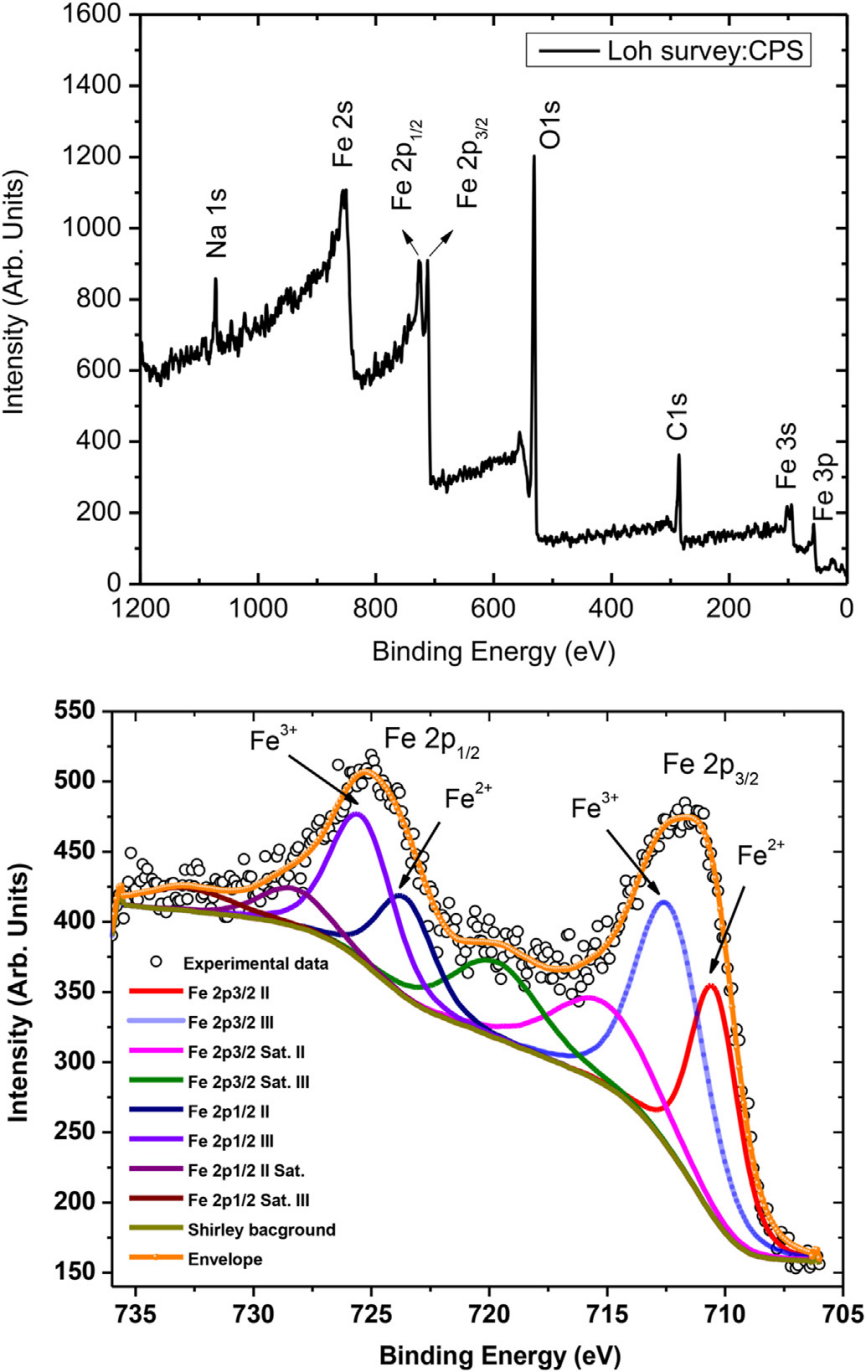
(a) Measured XPS spectrum of the medicinal grade Ayurveda Lauha Bhasma. (a) Survey XPS scan, (b) experimental and fitted XPS spectra for the characteristic spin orbit doublets of Fe.

To obtain a better understanding of the elemental composition and valence states of various constituent elements that co-exist in the Lauha Bhasma sample, X-ray photoelectron spectroscopy (XPS) measurements were carried out using synchrotron X-rays at BL-14 beamline [23] of the Indus-2 facility. The incident X-ray energy of 4.196 keV, monochromatized using a Si(111) double crystal monochromator, was used for sample excitation during the XPS measurements. The X-ray induced photo-electrons emitted from the Fe-Bhasma sample were collected and analyzed using a hemispherical analyzer equipped with an ultra low energy electron detection system (PHOIBOS 225, specs, Germany). During the measurements, the typical pressure in the beamline experimental station was maintained ∼5 × 10^−9^ mbar. The survey XPS spectrum of the Fe Bhasma sample (Fig. 2a) determines the presence of Na, O, and C elements. To identify different chemical states of Fe, the characteristic spin–orbit doublets located at the binding energy ranges of 710–712 eV and 724–727 eV, which correspond to the Fe 2p_3/2_ and Fe2p_1/2_, respectively, were analyzed.

After detailed fitting of the XPS experimental results (Fig. 2b), it was realized that each spin–orbit doublet peak is further composed of two component peaks corresponding to +3 and + 2 oxidation states of Fe. Our fitted results clearly showed that Fe^3+^ (Fe_2_O_3_) and Fe^2+^ (FeO) compounds coexist in the magnetite structure. These observations were found to be in good agreement with the previously reported work on Fe_3_O_4_ [24]. The fractional concentration ratio of Fe^2+^/Fe^3+^ ions in the Lauha Bhasma estimated from the XPS measurements was found to be 40%: 60%, which is slightly different from the theoretically expected values of 31%: 69% in the case of naturally occurring magnetite structure.

The transformation of bulk metallic iron into the Lauha Bhasma powder comprising of a nanoparticulate matter takes place via various oxidation and reduction processes that occur during different steps of the Ayurveda Bhasma preparation procedure. During the oxidation step solid iron reacts as follows in the presence of excess oxygen:

$$4Fe\left(Solid\right)+3O_{2}\left(gas\right)+6H_{2}O\left(liquid\right)\to 4Fe{\left(OH\right)}_{3}\left(porous\_rust\right)$$

The porous rust of iron finally transforms to the crystalline structure via following chemical (redox) reactions.

$$2Fe{\left(OH\right)}_{3}\overset{O_{2}}{\to }Fe_{2}O_{3}\left(Hematite\right)+3H_{2}O$$

Since it is already known that during the preparation of Ayurveda Bhasma various elaborated steps (e.g. *Bhanupaka*, *Putepaka* etc.) are strictly followed in a closed atmosphere/environment in the presence of carbon monooxide (CO) and carbon dioxide (CO_2_) gases, which are produced through the conventional route by burning dry cow dung. These gases allow reduction of the product material produced in earlier phases.

**Fig. 3:**
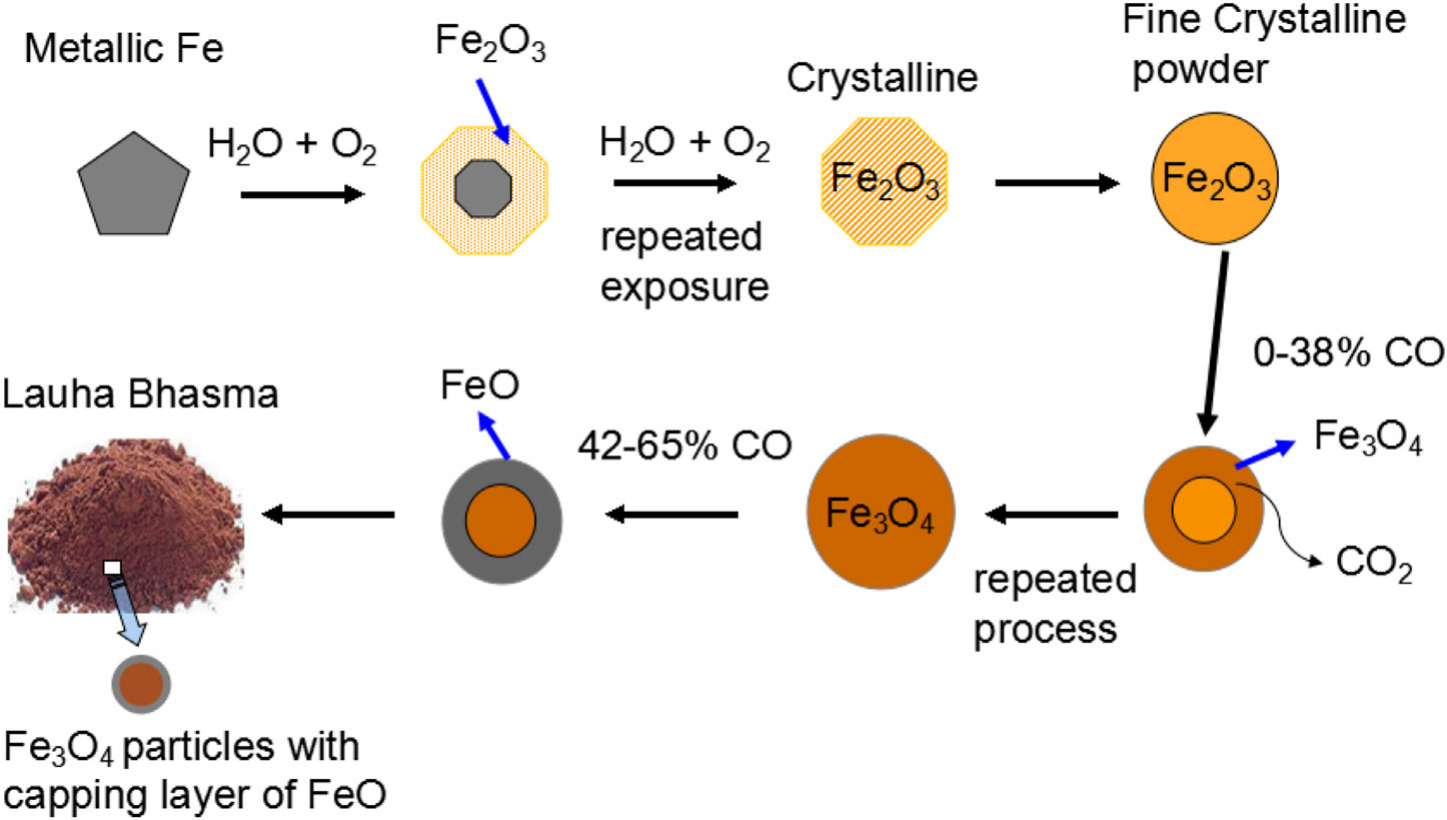
A schematic illustration of the probable oxidation and reduction mechanisms that occur during the preparation of Ayurveda Lauha Bhasma.

It is anticipated that in the case of the Lauha Bhasma, the reduction process of the hematite particles in the presence of carbon monooxide (CO) gas, most likely take place through the 2D-geometrical contraction model as shown in Fig. 3. It has previously been reported that the reduction mechanism in the case of Fe_2_O_3_ may be described by single rate-determining step and is contributed by a chemical reaction that primarily occurs at the particle surface [25,26]. The CO–CO_2_ gas mixture in varying ratios allows decoupling of the reduction of Fe_2_O_3_ particles into two distinct steps. The available literature suggests that at lower CO concentrations (0–38%), hematite (Fe_2_O_3_) particles reduce to magnetite (Fe_3_O_4_) structure, whereas at higher CO concentrations (42–65%), a layered structure of Wüstite (FeO) appears around magnetite particles in the form of an “egg” like structure. Finally, at the end of reaction one obtains Fe_3_O_4_ particles with a cover layer of FeO phase.

The 2D geometrical method as shown in Fig. 3, best describes the $Fe_{2}O_{3}\to Fe_{3}O_{4}$ and $Fe_{3}O_{4}\to FeO$ conversion processes. This clearly explains why specific procedures along with repeated steps are employed during the preparation of Ayurveda Lauha Bhasma in a closed environment. These processes are primarily responsible for slightly enhancing the Fe^2+^ content in the Lauha Bhasma particles as compared to that of the naturally occurring magnetite phase. This fact was clearly revealed from the XPS analysis.

### 3.2 Total reflection X-ray fluorescence measurements

In order to obtain information on the average particle size as well as the presence of any unwanted high-Z impurity elements, in case, if they are introduced in the Lauha Bhasm during the different steps of the preparation procedure, TXRF measurements were carried out using the BL-16 beamline of Indus-2 facility [27]. For this, Fe Bhasma sample was first dissolved into an ultra-pure water (Merck Millipore) medium and then dispersed ultrasonically. Approximately ∼20 μL volume of the diluted Fe Bhasma sample was then pipetted on top of a polished flat Si substrate.

**Fig. 4:**
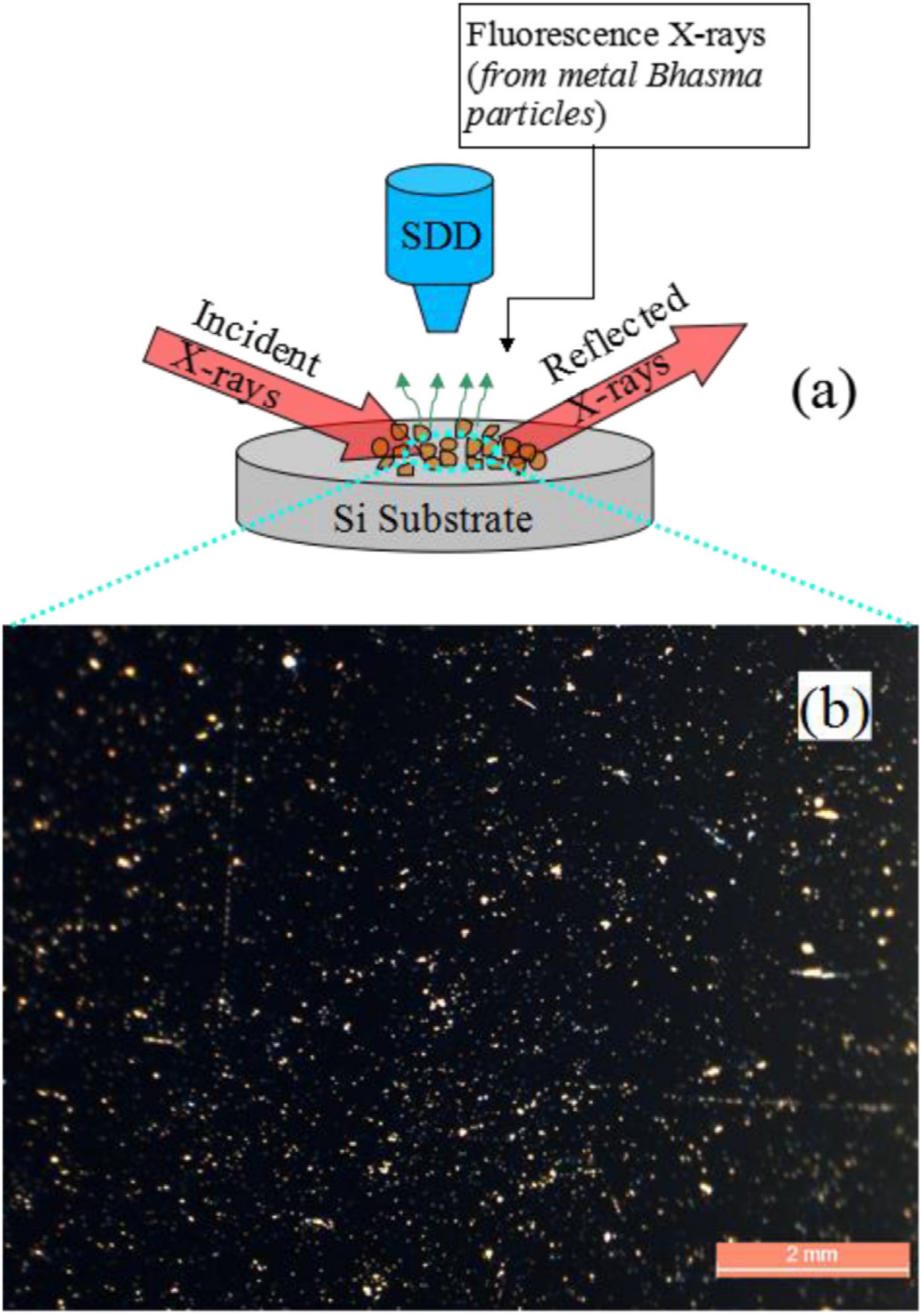
(a) Schematic of the TXRF measurement geometry used during the elemental analysis of Lauha Bhasma particles. (b) Measured optical micro-graph showing the dispersion of Lauha Bhasma particles on the Si substrate surface.

After a slow drying process at the room temperature, the residue of Fe Bhasma left over on the Si substrate surface (Fig. 4a), was directly investigated using the TXRF technique at an incident X-ray energy of 20 keV.

**Fig. 5:**
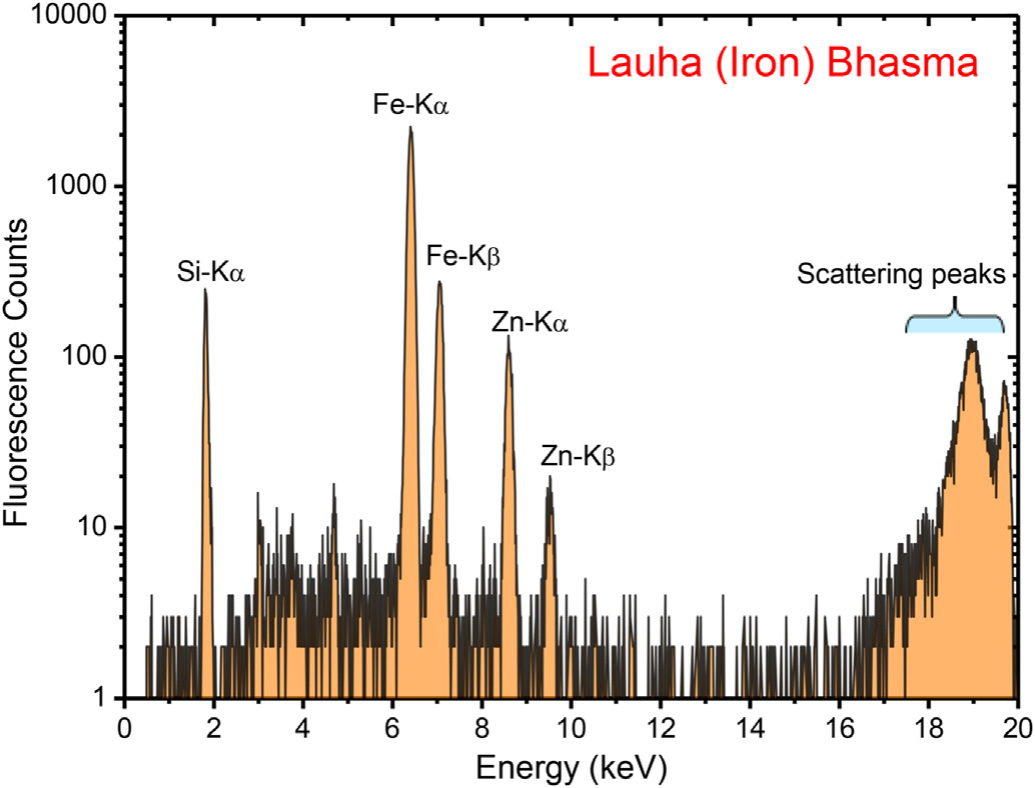
Measured TXRF spectrum of the particulate Lauha Bhasma sample at an excitation energy of 20 keV.

The measured TXRF spectrum of the Fe-Bhasma sample is shown in Fig. 5. The figure clearly shows the appearance of Fe-Kα and Fe-Kβ fluorescence lines primarily originating from the Fe-Bhasma sample. In addition, we also observed the presence of Zn in the Lauha Bhasma sample as an impurity element, which was found to be ∼50 ppm. Zinc is predicted to be introduced as an impurity in the Lauha Bhasma during any of the manufacturing stages or from other ingredients such as cow urine and Triphala powder, used during the synthesis of Fe Bhasma. However, the Si-Kα fluorescence peak originates from the Si substrate. The diluted Lauha Bhasma specimen deposited on the Si substrate was also analyzed using the optical measurements to view the dispersion of Fe particles on top of the Si surface. Fig. 4b shows the measured optical image of the Lauha Bhasma particles on top of the Si substrate. This figure shows that the Fe Bhasma particles on the Si surface are distributed uniformly.

### 3.3 Particle size measurements

**Fig. 6:**
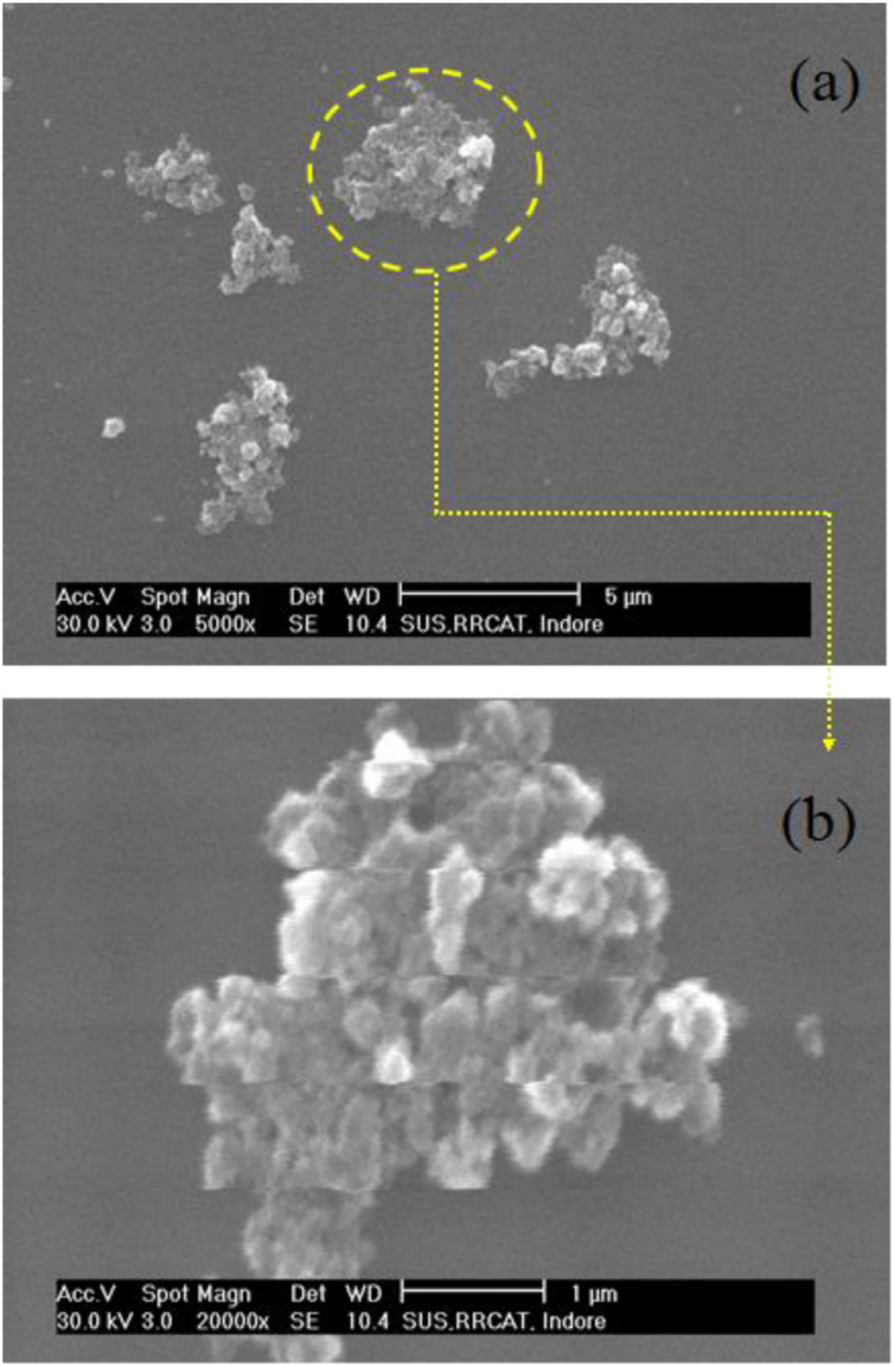
(a) Measured SEM of the Lauha Bhasma particle dispersed on top of a Si substrate surface. (b) Magnified image of an agglomerated particle showing clustering of individual nano-sized particles.

To further obtain the deep insights on the size distribution and shape of the Fe Bhasma particles we carried out scanning electron microscopy (SEM, Philips XL30CP) and grazing incidence X-ray fluorescence (GIXRF) measurements. Fig. 6 shows measured SEM images of the Fe particles dispersed on top of a Si substrate surface. It can be seen here that most of the Bshama's particles present in the form of agglomerates on the Si substrate surface whereas a few particles also appear in the form of single particulate matter. Fig. 6b depicts an expanded view of an agglomerated Fe Bhasma particle.

Here, it can be observed that an agglomerate of Fe Bhasma contains several smaller particles of sizes ranging from 100 to 800 nm. In order to further analyze the size distribution of Fe Bhasma particles, we carried out GIXRF simulations along with experimental measurements using monochromatic synchrotron X-rays of energy 10.0 keV.

**Fig. 7:**
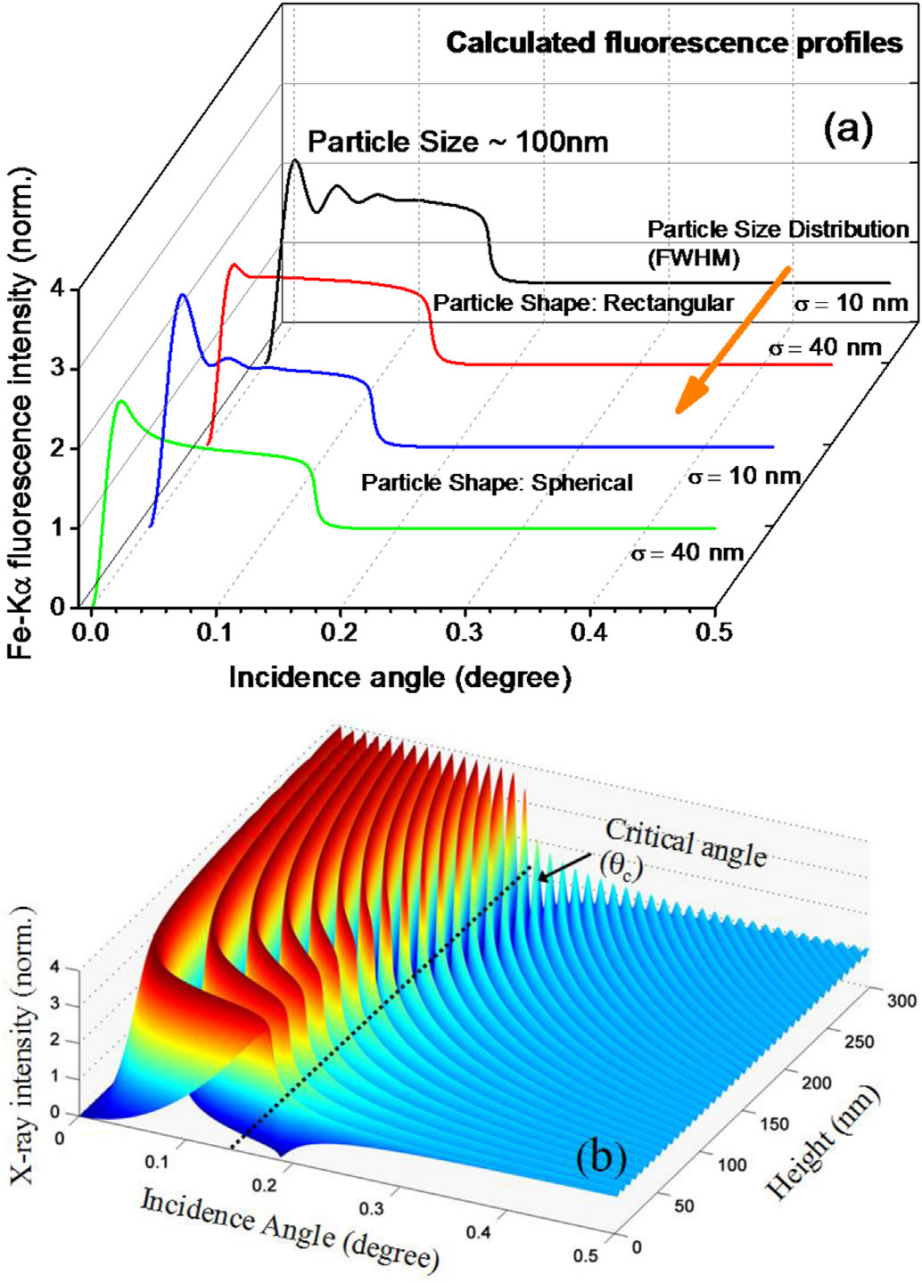
(a) Computed GIXRF profiles of Fe Bhasma particles assuming different r.m.s. deviations in the particle size distribution. These GIXRF profiles were computed considering the two distinct shapes (rectangular and spherical) of the particles. (b) Calculated E-field intensity distribution on top of the Si substrate surface at an X-ray energy of 10 keV.

**Fig. 8:**
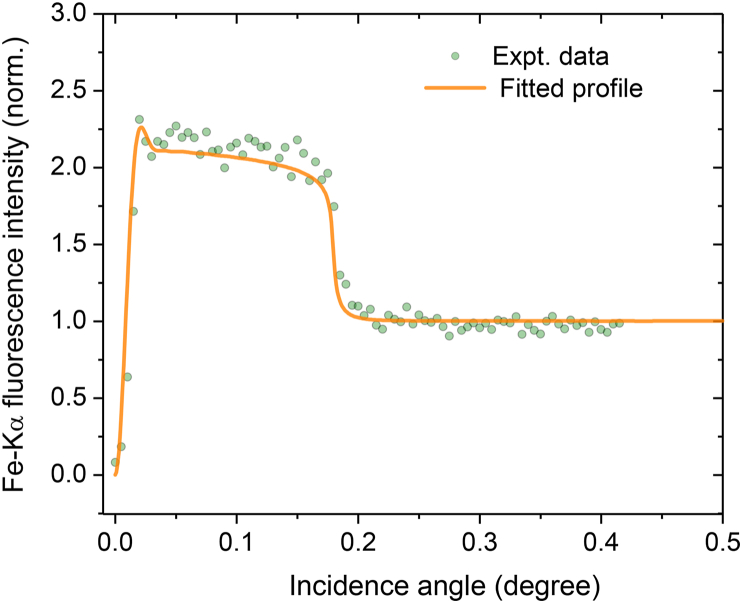
Measured GIXRF profile of the Lauha Bhasma particles as a function of grazing incidence angle Scattered points show the experimental data whereas solid line shows the fitted GIXRF profile.

Fig. 7a shows calculated GIXRF profiles for Fe-Kα fluorescence as a function of incidence angle assuming different shape and size distributions of Fe Bhasma particles. During the calculations, we have assumed an average particle size ∼100 nm with a root mean square (r.m.s.) deviation (σ) in the particle size distribution from 10 nm to 40 nm. During the computation we also considered two distinct shapes of the Fe Bhasma particles to see their effect on the observed GIXRF profile. From Fig. 7a, it can be clearly seen that the GIXRF profile is fairly sensitive to the particle size variation (σ) as well as the particle shape. These calculations clearly reflect that with the help of GIXRF investigations it possible to distinguish whether agglomerates of the Fe Bhasma particles are present in the form rectangular or spherical symmetry. In Fig. 8b, we have shown the calculated X-ray intensity distribution on top of the Si substrate surface at 10 keV incident X-ray energy. It can be seen here that below the critical angle (θ_c_ ∼ 0.179°), where total external reflection occurs (for 10 keV X-ray energy), the resultant X-ray intensity appears in the form of a well organized fringe pattern. This periodic pattern primarily arises due to the interference of incident and reflected X-ray wave fields below the critical angle region [28].

If a material comprising of particles with nano dimensions is excited with such an X-ray wave field distribution one observes intensity modulations in the measured GIXRF profiles (refer to Fig. 7a). In such a situation, the number of oscillations that appear in the measured GIXRF profile are fairly sensitive to the particle size distribution as well as on the shape of the particles. By carefully fitting the experimental data it is possible to determine the average size of the particles present in the Lauha Bhasma powder. In our case (Fig. 8), the determined average size of the Lauha Bhasma particles was found to be ∼100 nm with a r.m.s. variation in the particle distribution σ ∼40 nm, which was found to be in close agreement with reported literature [29]. Our results further showed that agglomerates of the Fe Bhasma particles on the Si surface mostly composed of rectangular shape. This fact was also clearly realized from SEM measurements.

### 3.4 Magnetic behavior of Lauha Bhasma

**Fig. 9:**
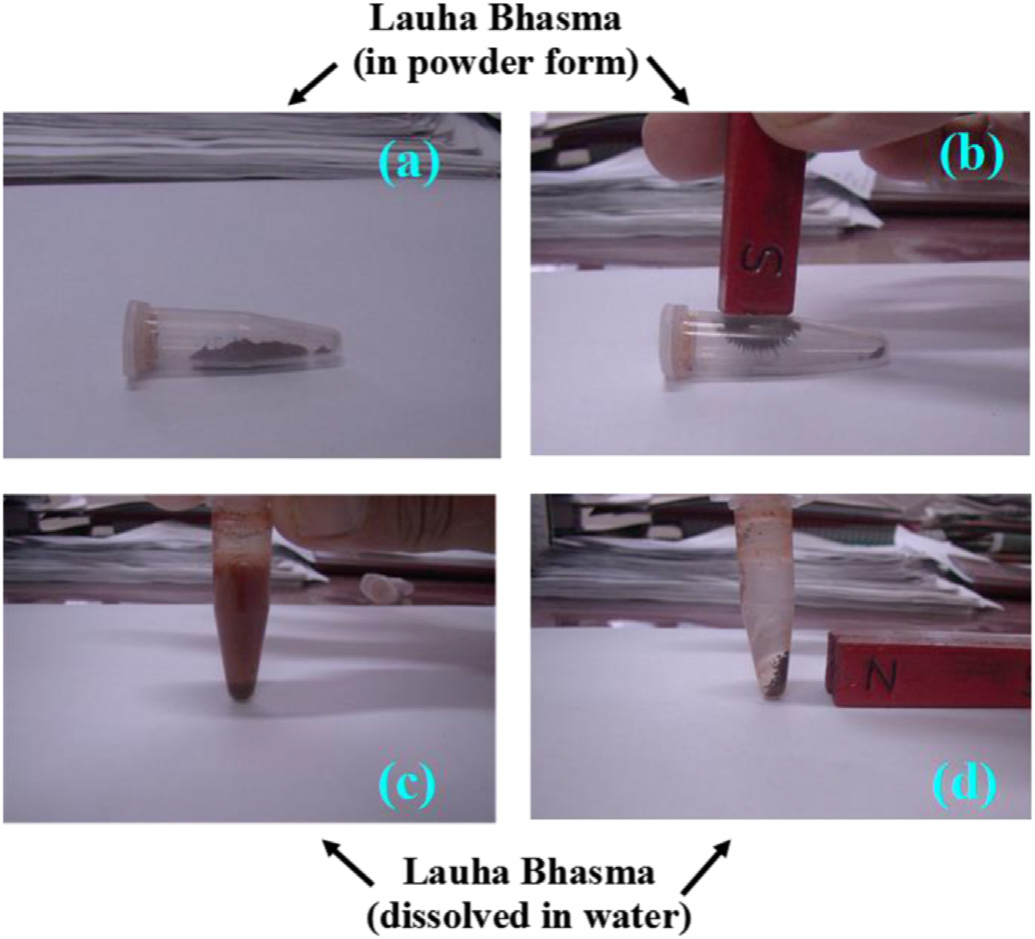
Magnetic behaviour of Lauha Bhasma particles in solid (a–b) and aqueous (c–d) media.

Lauha Bhasma particles have been found to be ferromagnetic in nature. When Lauha Bhasma particles are exposed to a weak external magnetic field in solid powder form, as well as when they are dissolved in water medium, they show strong attraction towards the external magnetic field. Fig. 9 depicts the magnetic behaviour of Lauha Bhasma particles in solid and aqueous media.

## 4 Discussion

The Iron metabolism of almost all living beings, including humans, is known to be highly complex. Its proper understanding is important for treating disorders caused mostly by iron overload or shortage in humans. Iron is an essential element of the heme group which allows binding of the oxygen (O_2_) in the lungs and its transport through the blood to different cells in our body. Except for iron in the heme group, there is no site for the transport and continuous supply of oxygen from the ambient air environment to a cell structure. Ferritin a is key protein found in our body that stores and regulates the availability of iron in the bloodstream. The iron in ferritin is primarily stored in the Fe^3+^ oxidation state. When our body demands iron, it is required that it be changed from the oxidation state of Fe^3+^ to Fe^2+^. Because Ayurvedic Lauha Bhasma contains both Fe(II) and Fe(III) oxidation states thus it may provide an obvious biogenic choice for our body to improve iron storage in the ferritin protein in the form of the Fe(III) state, as well as to maintain adequate availability of the Fe(II) state in the blood stream for better oxygen absorption in the lungs. Thus, it is expected that Lauha Bhasma may show better efficiency in treating anemia in humans as compared to that of the commonly used ferrous-based compounds in modern allopathy medicines. It has already been shown that magnetite nanoparticles are less toxic when their surface is modified or covered with a protective layer [30]. In this context, Lauha Bhasma particles may be of particular relevance for therapeutic applications due to the presence of an extra FeO covering layer on top of the magnetite particle.

Lauha Bhasma has a long history of routine use in the Indian Ayurvedic medicinal system for the treatment of anemia and other various diseases in humans. It can be considered a reasonably safe medicine due to the fact that its structure is found to be quite similar to the naturally occurring magnetite mineral. A recent experimental toxicity study conducted in albino rats reveals that the Lauha Bhasma do not show any deleterious effects even if administered dose is allowed to five times higher than the therapeutic dose [31]. The Lauha Bhasma may find its particular relevance in modern biomedical science because it can be effectively utilized as a carrier for targeted drug delivery and hyper thermia based cancer therapy applications [32]. In such a case, the heat produced by an oscillating magnetic field effectively causes necrosis of cancer cells without any noticeable damage to adjacent normal tissue [33]. It has been proposed that bio-endogenous magnetite minerals may play an important role in long-term information storage in the human brain and other species [34]. Aside from encouraging hemoglobin boosting activities, Lauha Bhasma in Ayurveda is also considered to offer anti-aging, memory rejuvenation benefits in humans.

## 5 Conclusions

To summarize, we have shown that modern synchrotron- based measurements are extremely valuable for investigating various Ayurveda drugs in order to establish their micro-structural properties, metallic nature, composition and chemical states of various elements present in them. The potential advantage of SR-assisted total reflection X-ray fluorescence spectroscopy is that it permits precise quantitative determination of various impurity elements present in a metal based Ayurveda drug if somehow they are introduced during the various preparation phases. Such analyses are extremely beneficial in gaining a thorough understanding of the physicochemical processes that evolved during ancient times for the preparation of various Ayurvedic medicines. It is also possible to correlate the necessity and effectiveness of these processes on human health in terms of currently available scientific knowledge. It has been observed that the medicinal grade Lauha Bhasma comprises of a slightly modified magnetite structure with a fractional percentage ratio of Fe^2+^/Fe^3+^ ions ∼40%: 60%. The average particle size of Lauha Bhasma was estimated to be 100 nm, with a standard deviation of 40 nm.

## Authors contribution statement

MK, conceptualized and designed the research. Arjun, prepared medicinal grade Lauha Bhasma samples for structural studies. MK, designed experiments and performed analyses. Ajay, performed XRF measurements. UK, carried out XPS experiments and analyses. MK, interpreted the data and wrote the manuscript. All authors approved the submitted version.

## Data availability statement

The raw data supporting the conclusions of this article will be made available by the authors, without undue reservation.

## Ethics statements

### Studies involving animal subjects

No animal studies are presented in this manuscript.

### Studies involving human subjects

No human studies are presented in this manuscript.

### Inclusion of identifiable human data

No potentially identifiable human images or data is presented in this study.

## Sources of funding

None.

## Declaration of competing interest

The authors declare that the research was conducted in the absence of any commercial or financial relationships that could be construed as a potential conflict of interest.
